# Deep Deterministic Policy Gradient-Based Resource Allocation Considering Network Slicing and Device-to-Device Communication in Mobile Networks

**DOI:** 10.3390/s24186079

**Published:** 2024-09-20

**Authors:** Hudson Henrique de Souza Lopes, Lucas Jose Ferreira Lima, Telma Woerle de Lima Soares, Flávio Henrique Teles Vieira

**Affiliations:** 1 Electrical, Mechanical and Computer (EMC) School of Engineering, Federal University of Goias (UFG), Goiânia 74605010, GO, Brazil; lima_lucas@discente.ufg.br (L.J.F.L.); flavio_vieira@ufg.br (F.H.T.V.); 2 Advanced Knowledge Center for Immersive Technologies (AKCIT), Federal University of Goiás (UFG), Goiânia 74605010, GO, Brazil; telma@inf.ufg.br

**Keywords:** deep reinforcement learning, network slicing, device-to-device, resource allocation

## Abstract

Next-generation mobile networks, such as those beyond the 5th generation (B5G) and 6th generation (6G), have diverse network resource demands. Network slicing (NS) and device-to-device (D2D) communication have emerged as promising solutions for network operators. NS is a candidate technology for this scenario, where a single network infrastructure is divided into multiple (virtual) slices to meet different service requirements. Combining D2D and NS can improve spectrum utilization, providing better performance and scalability. This paper addresses the challenging problem of dynamic resource allocation with wireless network slices and D2D communications using deep reinforcement learning (DRL) techniques. More specifically, we propose an approach named DDPG-KRP based on deep deterministic policy gradient (DDPG) with K-nearest neighbors (KNNs) and reward penalization (RP) for undesirable action elimination to determine the resource allocation policy maximizing long-term rewards. The simulation results show that the DDPG-KRP is an efficient solution for resource allocation in wireless networks with slicing, outperforming other considered DRL algorithms.

## 1. Introduction

Fifth-generation (5G), next-generation beyond 5G (B5G), and sixth-generation (6G) wireless communication networks are designed as multi-service networks to meet the diverse demands created by the emergence of demanding applications, such as smart cities, Internet of Things (IoT), and Industry 4.0. Recently, a large-scale dense network is growing as a trend for next-generation communication networks due to its advantages in terms of traffic capacity and diversified services. In this scenario, network slicing (NS) has received much attention as a flexible means of supporting service varieties. NS allows multiple independent and isolated virtual networks to coexist on the same network physical infrastructure [1].

Device-to-device (D2D) communication is also a promising solution for improving cellular network performance. D2D enables a direct connection between devices close enough with very little or no base station (BS) involvement. As such, it is a technique that can alleviate traffic from the BS. The user equipment (UE) in D2D communication has high transmission signal power and low transmission delay due to proximity. These characteristics result in increased spectral efficiency and throughput [2]. D2D communication is divided into two main categories: inband and outband. Each category is divided into two subcategories: underlay and overlay. In the inband category, D2D pairs use the frequency band of cellular communication, different from the outband category, which utilizes a different band. For the underlay subcategory, cellular and D2D communications share the cellular spectrum, while in the overlay subcategory, each D2D pair uses dedicated resources within the cellular spectrum [3].

Recently, the idea of combining D2D communication with NS in wireless network models has been on the rise [2]. Radio resource allocation effectively and efficiently in light of these paradigms is a complicated problem to solve; existing traditional approaches fail to provide good solutions due to the following characteristics [1]:

- Mathematical modeling: taking into account the diversity of network slice services combined with D2D communications and the constraints of the physical environment, it becomes complex to obtain a mathematical expression for the allocation of radio resources that meets the requirements of each service (e.g., latency, service-level agreement (SLA) and throughput).
- Traditional approaches do not adapt to episodic uncertainty: traditional optimization approaches require precise mathematical models with known parameters to adequately perform, which is often difficult to achieve in practice. Due to the lack of ability to explore and learn from the environment, traditional approaches frequently do not allocate resources efficiently.

In this context, deep reinforcement learning (DRL) algorithms, a branch of artificial intelligence (AI) that uses deep neural networks (DNNs), are attractive for exploring the environment and learning how to allocate radio resources. Algorithms using DRL have been considered excellent solutions for allocating radio resources in next-generation wireless networks that consider the NS paradigm [1,4,5].

This work proposes an approach named (DDPG-KRP) based on the deep deterministic policy gradient (DDPG) algorithm with K-nearest neighbor (KNN) clustering and reward penalization (RP). The KNN algorithm discretizes action at the output of the actor network that is part of the DDPG; the proposed reward penalization strategy consists of assigning a null reward if the action generated does not meet the SLA requirements of each slice. The proposed approach is based on improving agent learning and providing SLAs in each slice. To apply and evaluate the performance of the proposed approach through computational simulations, a mobile network scenario with D2D communication and network slices based on two service categories (ultra-reliable and low-latency communication (URLLC) and video) was considered. In this article, the underlay approach of the inband category is adopted to share the spectrum with all UEs connected to the D2D pair in each slice. We used the same data traffic model as in [1].

In summary, the main contributions of this paper are the following:

- The proposal of the DDPG-KRP algorithm applied to the problem of limited resource allocation from BS to UEs. The radio resource allocation problem is modeled through state transitions considering a mobile network with NS and D2D communication.
- A reward function is proposed in an augmented action space scenario where the agent learning process occurs without prior knowledge of a closed-form mathematical expression and system statistics, such as packet arrivals and channel quality of UEs. The proposed reward function generates a compromise between meeting the SLA requirements and maximizing the throughput and packet delivery ratio (PDR) in each slice while efficiently consuming the least amount of power possible. Reward penalty assigns power equal to zero to actions that do not satisfying the SLA constraints of each service.
- The action elimination strategy is integrated into the deep-Q network (DQN) and double DQN (DDQN) agent training to eliminate the combination of undesirable actions exceeding the total available resource capacity. Agent exploration is directed toward desirable actions, improving the chances of making the optimal resource allocation decision and increasing the training convergence speed.
- The DDPG-KRP algorithm performance is validated through extensive simulations. The results show that this algorithm significantly outperforms the other considered DRL-based methods due to better learning in high-dimensional action spaces.

The remainder of this paper is structured as follows: Section 2 presents the main works related to this study; Section 3 provides the formulation of the radio resource allocation problem in a network slicing scenario based on two service categories (i.e., URLLC and video), where each slice has a probability distribution representing its network traffic. This section also presents a reward function for the agent’s learning process; Section 4 begins with some reinforcement learning (RL) characteristics, then describes the motivation for the evolution of RL to DRL and introduces the proposed DDPG-KRP algorithm to solve the resource allocation problem considering NS and D2D and the strategy to reduce the high dimensionality of the action space; Section 5 discusses the results obtained from the DRL agents that learn through the reward function for resource allocation considering NS and D2D; and Section 6 summarizes the conclusions obtained.

## 2. Related Work

Radio resource management for improving the performance of next-generation wireless networks, such as those involving B5G, is a complex problem to solve using traditional mathematical methods. Therefore, considering related challenges, in the last decade, many research projects involving DRL, D2D, and NS have been developed, achieving promising results. In [6], the authors explored the sparse channel estimation (CE) problem of frequency-selective multipath channels in a filter bank multicarrier with offset quadrature amplitude modulation (FBMC/OQAM) industrial Internet of Things (IIoT) communication systems from the perspective of a sparse Bayesian learning (SBL) framework. The authors proposed the Block SBL (BSBL) algorithm to estimate the channel performance by exploiting the block–sparse structure of the sparse multipath channel model. Computer simulation results demonstrated the robustness of the BSBL CE approach in FBMC/OQAM systems, which can achieve lower mean square error (MSE) and bit error rate (BER) than the traditional least squares (LS) method and classical compressive sensing method.

An approach to allocating radio resources in networks that consider network slicing and D2D communication was presented in [2]. The authors presented an algorithm based on traditional mathematical methods that solved the NP-hard optimization problem to maximize the average spectral efficiency and throughput of the 5G heterogeneous cellular network based on D2D with NS. In other approaches that did not consider network slicing, the authors also formulated the resource allocation problem through a complex optimization problem. They used traditional algorithms to solve the problem, such as those described in the papers [3,7,8]. However, these approaches are not adaptive to the stochastic changes in the environment that consider network slicing and D2D communication. In this work, we propose using a DRL-based algorithm that adapts according to the environment variations and performs the adaptive joint allocation of power and bandwidth efficiently. Among the approaches that consider network slicing and D2D simultaneously, those that use reinforcement learning are the ones that have shown the best results [9]. Due to this reason, we focused on comparing the proposed DDPG-KRP algorithm with an approach using DQN that is similar to the one used in [4,9].

In [4], the authors investigated the application of DQN to solve some typical resource management problems in NS scenarios and demonstrated the advantages of this technique over some resource allocation schemes. The DRL agent allocated a bandwidth between slices, but a pre-training delay was found to satisfy the SLAs of each slice. In [1], similar to [4], the same probability distributions were used to represent the network traffic demand in each slice. However, in [1], the resource allocation problem was formulated as a constrained Markov decision process. To solve the problem, the authors proposed a DRL-based algorithm using interior-point policy optimization. The simulations, however, showed that the agent proximal policy optimization (PPO) acting without restriction can achieve good rewards. In this paper, the same network traffic demand was used for each slice and D2D communications were inserted for spectral efficiency optimization.

In [3], the authors formulated the resource virtualization problem in a wireless network with D2D communication underlay. This combination generated a nonlinear integer programming problem divided into two smaller linear integer problems. Both possible solutions were combined, and the problem was solved using two different schedulers. In [9], the authors proposed a resource allocation scheme between slices in a D2D-based virtualized communication network. A DRL agent was used for resource adjustment between slices. The authors formulated the resource allocation problem as a convex optimization problem and solved it with an alternating direction method. They aimed to balance resource utilization and quality of service (QoS) for multiple slices. In [10], the authors proposed an energy-efficient resource allocation model for D2D communication in wireless personal networks. The algorithm is compared with other models in the literature and shows good results in the proposed scenario without network slicing.

In [11], the authors proposed a DRL-based NS technique. The DQN algorithm was more specifically used to find the resource allocation policy maximizing the throughput while satisfying the QoS requirements in B5G systems. The authors employed a technique for eliminating undesirable actions that cannot satisfy the QoS requirements. Accordingly, the DRL agent exploration was directed toward desirable actions, improving the chances of making the optimal decision in resource allocation and enhancing the convergence speed in DRL training. The output of the DQN neural network is based on the discrete amount of possible actions; hence, a huge action space makes it difficult to learn the agent. In this work, we approach the DDPG agent with KNN to take the best action in a discrete space. In addition, we propose a reward penalization approach for the DDPG algorithm so that the agent does not take undesirable actions, to try to efficiently solve the problem of power and bandwidth allocation in networks with network slicing and D2D communication. In [12], the authors proposed a DRL-based approach for inter-slice resource allocation in a dense long-range wide-area network. Each slice has a corresponding DQN agent that allocates the spread factor and the transmission power to the IoT devices to provide the QoS requirements. In other words, the conventional scheme was replaced with multi-agent DRL with different reward function designs for each slice according to the QoS requirements. In this work, a scenario is assumed considering massive device connection in a small cell and a bandwidth of 10 MHz, similar to that in [3,4,9,13]. One of the pillars of an IoT network based on intelligent sensors for industrial applications is the efficient allocation of available radio resources. Resource management in wireless networks has become an increasingly complex demanding solution that has adapted to the non-trivial nature of the current difficulties. In this work, DRL algorithms are used to find solutions for the resource allocation problem in systems involving a massive connection of intelligent devices.

In [14], the authors proposed an adaptive NS algorithm for vehicular networks to temporarily prioritize emergency traffic in emergency situations while maintaining an acceptable QoS for non-incident-related services in a radio resource-constrained environment. Software-defined networking (SDN), network function virtualization (NFV), and fog computing paradigms mainly enabled the proposed solution. However, the authors reported the need to have a DRL method to support the vehicular application module in resource allocation between slices. In this work, our main objective is to solve the problem of allocating radio resources flexibly and efficiently, improving the SLA required for each service. To achieve this goal, we propose the use of D2D communication in conjunction with NS. The task of radio resource allocation in this scenario is a challenge even for intelligent algorithm models based on deep reinforcement learning such as DQN and DDQN because the output of the neural network of these agents is based on the number of possible combinations of actions, which in large-scale mobile network environments leads to the problem widely known in the literature as the curse of dimensionality [15]. In this work, we propose using the DDPG algorithm because the action this agent takes does not start from a choice of action from the total number of possible combinations, i.e., the search space is adequately reduced, making the DDPG algorithm more efficient. We present an advance in the state of the art with the proposal of the DDPG-KRP algorithm, inserting the KNN algorithm into the DDPG agent to discretize the action taken, and using the reward penalty to avoid choosing undesired actions.

## 3. Resource Allocation Considering NS and D2D

**Figure 1:**
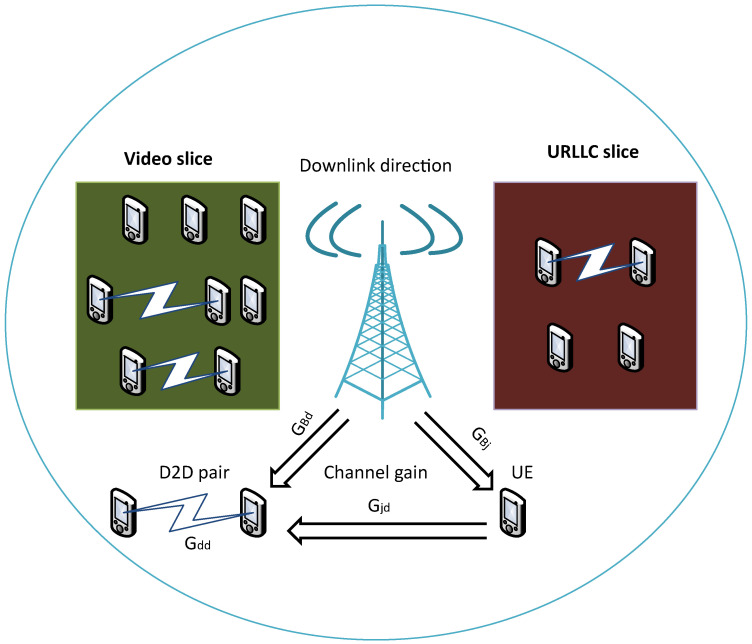
Mobile communication system model.

For the sake of readability, all the symbols and optimization variables are summarized in Table 1. This section discusses the state and action spaces and the reward function for the proposed scenario. We considered that a single BS provides the radio resource for network slices running different services (i.e., URLLC and video). D2D communication occurs in each slice of the network, as shown in Figure 1. The considered wireless communication system model refers to the downlink direction and comprises a small cell with a BS at its center. We used the inband category and the underlay subcategory to reuse the spectrum while considering the interference effect on the resource allocation to any UE connected to the D2D pair. However, we assume that UEs do not reuse spectra from other UEs.

Similar to that in [1], the traffic demand of the UEs was generated by using the probabilistic distributions described in Table 2 based on their respective streaming model. Accordingly, in this work, resource allocation is performed at each transmission time interval (TTI), and orthogonal frequency division multiplexing (OFDM) is considered the technique utilized in the long-term evolution (LTE) transmission downlink, which allows a simultaneous transmission of different data packets by assigning different sub-carriers to the user [16]. A resource block (RB) is the minimum resource allocation and includes resources in the time and frequency domains. The composition of an RB in 5G mobile networks is more flexible based on its numerology; in this work, we have assumed numerology 0, i.e., in the frequency domain, it is composed of 12 consecutive 15 KHz sub-carriers, and in the time domain, it has a duration slot of 0.5 ms [15]. In our implementation, we consider a bandwidth of 10 MHz. In such a system, we have 50 RBs per slot. RBs are always scheduled in pairs composing a scheduling block (SB), with a duration of 1 ms [16].

### 3.1. Channel Model

In this work, the bandwidth allocated to the D2D pair is the same as the UEs connected to the D2D pair. The spectrum between the UEs and the connected D2D pairs is reused; hence, the total throughput of the network slice is increased. When packet transmission occurs in the downlink direction, UEs are exposed to interference when any D2D peer transmits using the same allocated bandwidth. Our focus in this work is on a single cell scenario. Therefore, we do not consider intercell interference. When two devices communicate using the same frequency band, there is the possibility of interference. This interference can reduce the quality of communication between these devices or even lead to a total loss of communication. One of the main challenges of D2D communication is managing this interference [10].

The interference from the D2D communication to the UEs in the cellular network must be restricted to maintain a minimum level of network performance. If the distance between the D2D devices is long, the D2D will require more power. In this case, the cellular network loses performance because of interference [17]. The interference level will not only depend on the transmission power of the D2D pair but also channel gain between the D2D pair and the UEs. The signal to interference plus noise ratio (SINR) of UE *j* is given as follows:

(1) $$\begin{array}{c} \Gamma_{j}=\frac{p_{j}\times G_{Bj}}{\sigma^{2}+\sum_{d=1}^{D}p_{d}\times G_{jd}}, \end{array}$$

where $G_{jd}$ denotes the channel gain between the UE *j* and the D2D pair *d*, $G_{Bj}$ denotes the channel gain between the BS and the UE *j*, $p_{j}$ and $p_{d}$ are transmission signal power from UE *j* and the D2D pair *d*, respectively. $\sigma_{z}^{2}$ is the noise power. The SINR of the D2D pair *d* is given as

(2) $$\begin{array}{c} \Gamma_{d}=\frac{p_{d}\times G_{dd}}{\sigma^{2}+\sum_{j=1}^{Z}p_{j}\times G_{Bd}}, \end{array}$$

where $G_{dd}$ denotes the channel gain between the D2D pair *d* devices, and $G_{Bd}$ denotes the channel gain between the BS and the D2D pair *d*. According to [17], the channel gain between two devices is represented as

(3) $$\begin{array}{c} G_{uv}=K_{uv}\times d_{uv}^{-\delta }, \end{array}$$

where $d_{uv}$ is the distance between the transmitter *u* and the receiver *v*; δ is the constant path loss exponent; and $K_{uv}$ is a normalization constant that depends on the propagation properties of the environment. In this work, we assumed that δ = 2 and K=0.001. The linear gain between the BS and the UE is given as follows:

(4) $$\begin{array}{c} G_{Bj}=10^{-PL/10}, \end{array}$$

where PL is the path loss received by the small cell. The Okumura–Hata model was used to predict path loss [2]:

(5) $$\begin{array}{c} PL=140.7+36.7\times log(dist[\mathrm{km}])\;\mathrm{dBm}, \end{array}$$

where dist is the distance between the UE and the BS. The achievable data rate of D2D pair *d* can be obtained by Shannon capacity:

(6) $$\begin{array}{c} C=w_{d}\times log_{2}\left(1+\Gamma \right), \end{array}$$

where $w_{d}$ is the bandwidth of the D2D pair shared with the UE.

### 3.2. System Model and Problem Formulation

The water-filling method aims to allocate the BS signal power to the UEs in such a way as to maximize the sum of the network data rate and meet the constraint of allocating power with positive values to the UEs, satisfying the BS power availability [18]. The water-filling method was chosen because it solves the optimization problem described by Equations (7)–(9) with linear computational complexity by solving a system with (*Z* + 1) equations and (*Z* + 1) unknowns.

(7) $$\max_{p}\;\sum_{j=1}^{Z}w_{j}\times log\left(1+p_{j}\times \frac{G_{Bj}}{\sigma^{2}+\sum_{d=1}^{D}p_{d}\times G_{jd}}\right),$$

(8) $$\mathrm{Subject}\;\mathrm{to}\;\sum_{i=j}^{Z}p_{j}\leq P_{bs},$$

(9) $$p_{j}\geq 0,\;i=1,2,\ldots ,Z.\;P_{bs}\geq 0,$$

where *Z* is the total number of UEs, $w_{j}$ is the bandwidth allocated to the UE *j*, $P_{bs}$ is the BS power and $p_{j}$ = $p_{1},p_{2},\ldots ,p_{Z}$ is the power allocated to the UEs. The expression $\frac{G_{Bj}}{\sigma^{2}+\sum_{d=1}^{D}p_{d}\times G_{jd}}$ represents the channel state information (csi). By applying Lagrange’s dual method (L), the objective function (7) can be reduced to

(10) $$\begin{array}{c} L\left(p,\lambda \right)=\sum_{j=1}^{Z}w_{j}\times log\left(1+p_{j}\times \frac{G_{Bj}}{\sigma^{2}+\sum_{d=1}^{D}p_{d}\times G_{jd}}\right)-\lambda \left(\sum_{j=1}^{Z}p_{j}-P_{bs}\right), \end{array}$$

where λ is the Lagrange multiplier. A solution to the dual problem in Equation (10) is obtained by setting the gradient to zero, i.e., ∇L(p,λ) = 0, as follows:

(11) $$\begin{array}{ccc} \frac{\partial L(p,\lambda )}{\partial p} & = & \frac{\frac{G_{Bj}}{\sigma^{2}+\sum_{d=1}^{D}p_{d}\times G_{jd}}}{1+p_{j}\cdot \frac{G_{Bj}}{\sigma^{2}+\sum_{d=1}^{D}p_{d}\times G_{jd}}}-\lambda =0, \end{array}$$

(12) $$\begin{array}{ccc} p_{j} & = & \frac{1}{\lambda }-\frac{1}{\frac{G_{Bj}}{\sigma^{2}+\sum_{d=1}^{D}p_{d}\times G_{jd}}}. \end{array}$$

Therefore, the power allocation for all UEs can be rewritten as follows:

(13) $$\begin{array}{c} p_{j}\left(t\right)=\max\left\{0,\frac{1}{\lambda_{t}}-\frac{\sigma^{2}+\sum_{d=1}^{D}p_{d}\times G_{jd}}{G_{Bj}}\right\},\forall j=1,2,\ldots ,Z. \end{array}$$

**Figure 2:**
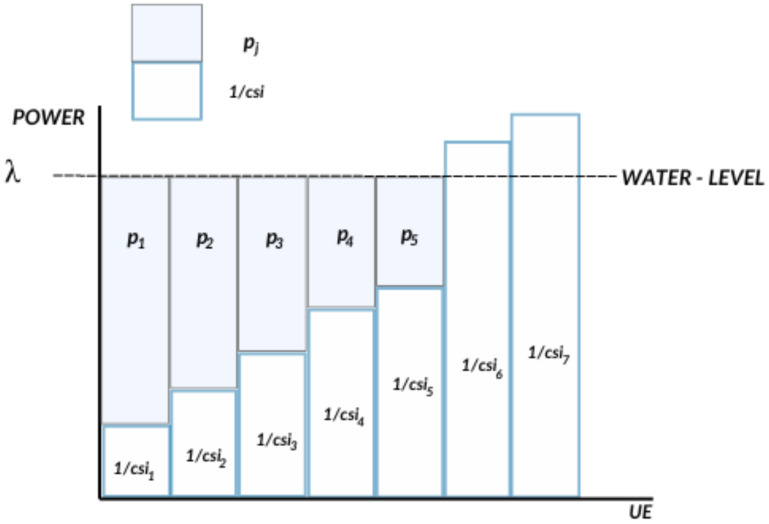
Power allocation using the water-filling algorithm.

The choice for the λ value determines the “water level” for the constraint of the sum of power allocated to the UEs, i.e., the total power consumed by the BS as shown in the Figure 2. The algorithm considers it unnecessary to allocate power to some UEs given the considerably high noise level, by limiting communication only to UEs with better channel quality so that the data rate is as high as possible. This mechanism is especially crucial for providing some QoS requirements in wireless networks.

Traditional approaches to solving the water-filling method consist of choosing the best lambda value for allocating power to UEs to maximize throughput. In a modern wireless network scenario with network slicing and D2D communication, the power allocation problem is not only concerned about maximizing total throughput, but it also becomes a part of the resource allocation, whose main objective is to meet the SLA requirements of each slice. The choice of λ value, the main parameter for power allocation, is made by an intelligent agent according to a reward function. Using the value of λ, we apply the water-filling algorithm to allocate power to the UEs.

In this paper, we propose modeling the problem of power and bandwidth allocation through state transition, where the RL agent decides which action to take by exploring the environment and maximizing the long-term reward [19]. The RL environment is represented by three components, state, action, and reward, which are described below:

State: The system state s(t) is represented by the number of packets received NPR in each slice *i* and by the csi each UE *j*.

(14) $$\begin{array}{c} s\left(t\right)=\left\{NPR_{i},csi_{j}\right\}.\;\forall i\in F,\;\forall j\in Z. \end{array}$$

Action: The action is given by the bandwidth *w* allocation to each slice *i* and by the power allocation using Equation (13) through the λ value, which is discretized in two decimal places ∈ (0.01, 1). The bandwidth is discretized in slots and can take discrete values in the range of 1 to 2000 slots for a bandwidth total of 10 MHz in a time of 1 s.

(15) $$\begin{array}{c} a\left(t\right)=\left\{w_{i},\lambda \right\}.\;\forall i\in F. \end{array}$$

Reward: We propose that the reward function r(s(t),a(t)) be given by the weighted sum from the Shannon channel capacity (*C*), by the package delivery rate (PDR) satisfaction index to each slice and by the power allocated to UEs ($p_{j}$). The PDR is obtained by dividing the number of packets transmitted (NPT) by the total number of packets received (NPR). The reward penalty occurs if the chosen action does not meet the SLA requirements or exceeds the BS’s total available resources, as follows:

(16) $$r\left(s\left(t\right),a\left(t\right)\right)=\left\{\begin{array}{c} 0,\mathrm{if}\;\mathrm{the}\;\mathrm{action}\;\mathrm{chosen}\;\mathrm{is}\;\mathrm{undesirable}\;\\ \sum_{i=1}^{F}\left(\alpha \times C_{i}\right)+\beta \times \left(PDR_{i}-\sum_{j=1}^{Z}p_{j}\right), \end{array}\right.$$

where α and β are constants used to add weight to values and maintain them with the same number of digits. The SLAs impose stringent requirements on the PDR [1]. Generally, RL agents learn with the main objective of maximizing the reward function. In this work, the main objective is to reduce the least amount of signal transmission power possible; the reward function is negative in the equation, thus minimizing its value.

### 3.3. Network Energy Efficiency

The power allocated to the UE is a crucial factor that directly influences the quality of the communication channel. However, UEs communicating with D2D pairs using the same channel resources creates the possibility of interference. This interference can reduce the quality of the communication and even lead to a total loss of communication. Therefore, power transmission control can be used to reduce the power of the interfering device, which will reduce interference and improve communication quality [10].

D2D communication can reduce the total power consumption of UEs while providing a reliable and efficient communication service. One metric by which efficient power consumption can be assessed is energy efficiency, which in this work is given by the ratio of total throughput to total power consumed, given by the equation below:

(17) $$\begin{array}{c} EE=\frac{\sum_{j=1}^{F}C_{j}+\sum_{d=1}^{D}C_{d}}{\sum_{j=1}^{Z}p_{j}}. \end{array}$$

### 3.4. End-to-End Delay

End-to-end delay can be caused by a variety of factors, including optimal path selection, queue length, and communication period. The delay is calculated based on the time *t* that the packets remain in the queue waiting for transmission. We assume a first-in-first-out queue satisfying the SLA constraints in each service of Table 2. The traffic that cannot be consumed in the current slot is queued to the next slot represented by the queuing equation, given by

(18) $$\begin{array}{c} NPQ(t)=NPT(t)-NPR(t)+NPQ(t-1), \end{array}$$

where NPQ(t) is the number of packets in the queue at time *t*.

## 4. Deep Reinforcement Learning-Based Algorithms

This study considered reinforcement learning algorithms, in which the agent interacts with the environment in discrete decision epochs. At each decision epoch *t*, the agent observes the state s(t), takes action a(t) based on its policy π(s), and receives a reward r(s(t),a(t)). The environment transitions into the next state s(t+1). The goal is to find the optimal policy $\pi {\left(s\right)}^{*}$ for mapping states to actions, which maximizes the discounted cumulative reward, given as follows [5]:

(19) $$\begin{array}{c} R=\sum_{t=0}^{T}\gamma \times r\left(s\left(t\right),a\left(t\right)\right), \end{array}$$

where the discount factor γ∈[0,1) is a constant that deducts future rewards, and *T* represents how many time steps are still left in the training session or episode [5].

The Q-learning algorithm is based on searching for tabular values, and because the space of states and actions in this work is large, it takes a long time to converge to a good solution [19]. Thus, our work exploits the DQN; with DQN, the Q-value function Q(s(t),a(t)) is approximated by a deep neural network (DNN), different from Q-learning where the Q-value function is tabular values. DNNs are exploited to accelerate Q-learning convergence and improve its generalization capability in new states. The DNN is denoted by Q(s(t),a(t)|Θ), where Θ represents its parameters or weights. Another neural network is used to increase the learning stability of the DQN. It is called the target Q-network, and its weights $\Theta^{\prime }$ are periodically updated to follow the weights of the main Q-network [20]. The DQN algorithm is optimized iteratively, updating the weights Θ of its neural network to minimize the Bellman loss function as follows [20]:

(20) $$\begin{array}{c} L\left(\Theta_{t}\right)=E_{s\left(t\right),a\left(t\right),r_{t},s\left(t+1\right)}[r_{t}\left(s\left(t\right),a\left(t\right)\right)+\gamma \times \max_{a(t+1)}Q\left(s\left(t+1\right),a\left(t+1\right)|\Theta^{\prime }\right)\\ -Q\left(s\left(t\right),a\left(t\right)|\Theta \right){]}^{2}. \end{array}$$

where *E*[·] is the expectation operator.

The DQN algorithm tends to overestimate Q-values. This is because it uses the same training transitions for action selection and evaluation, which can degrade the training process and lead to sub-optimal policies. To solve the problem, we used the DDQN technique, which employs two functions for the Q-value: one to select the best action and the other to evaluate this action. The action selection is still based on the Θ weights. The second $\Theta^{\prime }$ weights are used to evaluate the policy value. The DDQN algorithm uses the following modified Bellman loss function to update its weights [20]:

(21) $$\begin{array}{c} L\left(\Theta_{t}\right)=E_{s\left(t\right),a\left(t\right),r_{t},s\left(t+1\right)}[r_{t}\left(s\left(t\right),a\left(t\right)\right)+\\ \gamma \times Q\left(s\left(t+1\right),\arg\max_{a(t+1)}Q\left(s\left(t+1\right),a\left(t+1\right)|\Theta \right),\Theta^{\prime }\right)-Q\left(s\left(t\right),a\left(t\right)|\Theta \right){]}^{2}. \end{array}$$

### 4.1. Deep Deterministic Policy Gradient Algorithm

In [21], the DDPG was proposed by integrating DQN and the actor–critic method to solve problems with a continuous, high-dimensional action space. DDPG is one of the agents that perform policy learning using the gradient of the objective function (22) that represents the expected long-term reward. The DDPG agent learns a policy that is continuous and deterministic. To guarantee the exploration of the environment, the trajectories generated by the agent come from another policy, which is stochastic and makes DDPG an off-policy method. The space of observations can be discrete or continuous, but the space of actions has to be continuous. The DDPG algorithm is model-free, i.e., it does not need the model of the environment, and to perform learning, it learns both the deterministic policy $\mu (s|\theta^{\mu })$, parameterized by $\theta^{\mu }$, and the value function of the action–state pair for this policy $Q(s,a|\theta^{Q})$. Therefore, it uses actor–critic architecture.

(22) $$\begin{array}{c} \nabla_{\theta^{\mu }}J\approx E\left[\nabla_{\theta^{\mu }}Q\left(s\left(t\right),a\left(t\right)|\theta^{Q}\right){|}_{s=s\left(t\right),a=\mu (s\left(t\right)|\theta^{\mu })}\right]. \end{array}$$

The authors in [22] show that the gradient of this objective function concerning the parameters $\theta^{\mu }$ is given by:

(23) $$\begin{array}{c} \nabla_{\theta^{\mu }}J\approx E\left[\nabla_{a}Q\left(s\left(t\right),a\left(t\right)|\theta^{Q}\right){|}_{s=s(t),a=\mu (s(t))}\cdot \nabla_{\theta^{\mu }}\mu \left(s\left(t\right)|\theta^{\mu }\right){|}_{s=s(t)}\right]. \end{array}$$

**Figure 3:**
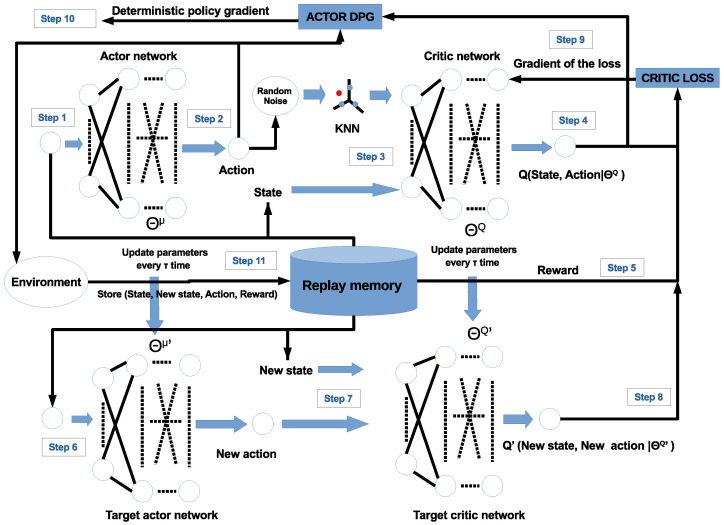
DDPG-KRP architecture, the red and blue circles represent the actions in continuous and discrete space, respectively.

Note that the equation above is in the format of the actor–critic architecture, where the actor is related with $\nabla_{\theta^{\mu }}\mu \left(s\left(t\right)|\theta^{\mu }\right)$ and the critic is related with $\nabla_{a}Q\left(s\left(t\right),a\left(t\right)|\theta^{Q}\right)$. The actor–critic method consists of a neural network, called the actor, and another, called the critic. The actor network takes the state as its input and produces a value for the action, while the critic network is a Q-value network that takes both state and action as the input and produces the Q-value as a single output. As in the DQN, the DDPG also uses target networks that are time-delayed copies of their original networks, which slowly follow the learned networks. Another DQN feature used in the DDPG is the replay buffer ($R_{b}$) for the experiment sample to update the neural network parameters, as shown in Figure 3. Figure 3 shows the learning process of the DDPG-KRP algorithm in steps.

In Algorithm 1, the parameters of the copies are updated periodically at every τ time in a smoothed manner concerning the originals through a first-order filter with a smoothing factor (ρ) of $10^{-5}$. These copies are called target networks. Using these target network values greatly improves the learning stability [20]. Algorithm maintains a parameterized actor function, $\mu (s|\theta^{\mu })$, that specifies the current policy, deterministically mapping states to a specific action. The critic network $Q(s,a|\theta^{Q})$ learns using the Equation (24). The actor is updated following the chain rule applied to the expected return from the start of the *J* distribution as Equation (23) [21,23].

(24) $$\begin{array}{c} L=\frac{1}{N}\sum_{i=1}^{N}{\left(y\left(i\right)-Q\left(s\left(i\right),a\left(i\right)|\theta^{Q}\right)\right)}^{2}, \end{array}$$

where y(i) for i=1,…,N are the desired output values for the training input samples given by the Bellman loss function, i.e., $y\left(i\right)=r\left(i\right)+\gamma \times Q^{\prime }\left(s\left(i+1\right),\;\mu^{\prime }\left(s\left(i+1\right)|\theta^{\mu^{\prime }}\right)|\theta^{Q^{\prime }}\right)$ where $\mu^{\prime }\left(s\left(i+1\right)|\theta^{\mu^{\prime }}\right)$ and $Q^{\prime }\left(s\left(i+1\right),\mu^{\prime }\left(s\left(i+1\right)|\theta^{\mu^{\prime }}\right)|\theta^{Q^{\prime }}\right)$ are the target networks of the actor and the critic, respectively.

Since DDPG generates a deterministic policy, to ensure the exploration of the environment, it was proposed to add a noise (η) in the actor output. In this case, the output of the actor network is given by

(25) $$\begin{array}{c} a\left(t\right)=\mu \left(s\left(t\right)|\theta^{\mu }\right)+\eta_{t}. \end{array}$$

**Algorithm 1:** DDPG-KRP
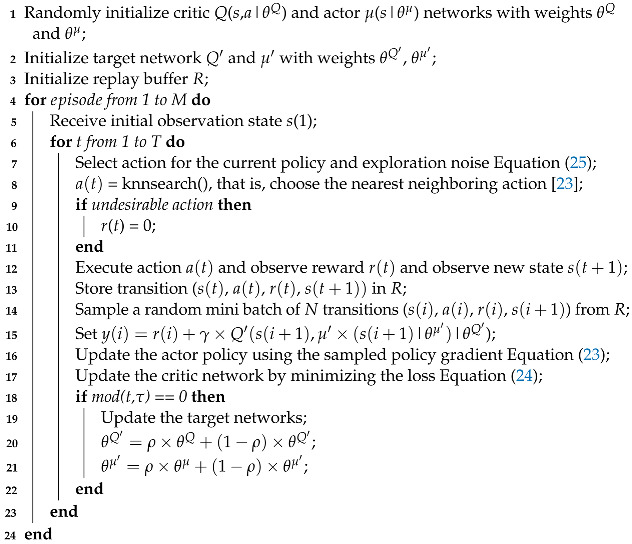



In this paper, we propose using the Ornstein–Uhlenbeck process that generates a temporally correlated noise of zero mean [24] during the training phase to balance the exploration. The initial value of the standard deviation of the noise is 0.5, and the standard deviation decay rate used is 0.01 per update step. The target parameters in both actor and critic networks are updated with soft updates as follows:

(26) $$\begin{array}{c} \theta^{\mu^{\prime }}=\rho \times \theta^{\mu }+\left(1-\rho \right)\times \theta^{\mu^{\prime }}. \end{array}$$

(27) $$\begin{array}{c} \theta^{Q^{\prime }}=\rho \times \theta^{Q}+\left(1-\rho \right)\times \theta^{Q^{\prime }}. \end{array}$$

### 4.2. K-Nearest Neighbors and Reward Penalization

**Figure 4:**
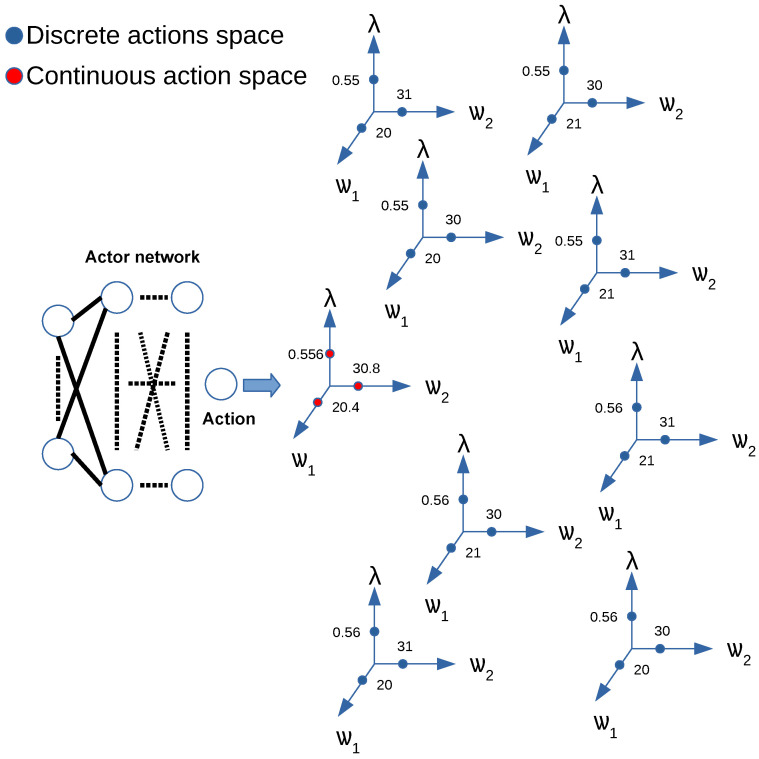
KNN algorithm applied to discretize the value of the action generated at the output of the actor neural network.

DDPG was originally designed to handle continuous action spaces. However, resource allocation is a discrete decision problem where the combination of the resource allocation cannot exceed the available total value. Inspired by the approach in [15], we use the KNN to discretize DDPG to adapt it to discrete action spaces. The basic idea is to use a continuous-based algorithm to generate an initial continuous action first. Then, the K nearest discrete actions are found using the KNN algorithm [23]. We chose the nearest neighboring action, as shown in Figure 4.

In terms of computational overhead, the DDPG algorithm has a better training performance because the action generated at the output of its actor neural network is related to only one neuron, while the other methods have one neuron at the output of their neural network for each possible combination of actions, which in our scenario amounts to approximately 500,000 combinations. Therefore, our proposed approach that uses the KNN algorithm improves the performance of the DDPG-KRP algorithm as well as the efficiency of the DDPG considering large networks. Several recent works have attempted to solve the problem of some RL approaches presenting a large discrete action space by discretizing the continuous action space [15,25]. In this sense, as shown by the proposed Algorithm 1, a KNN approximation is used because of its agile search in logarithmic time and maps a continuous action space to a discrete action space [15,23]. We propose applying a method of eliminating undesired actions to the DDPG agent that consists of penalizing the reward function, i.e., during the training phase, if the actor–network generates an action that does not satisfy the SLA constraints and exceeds transmission power available, the reward will be a value of 0.

This work presents an advance by proposing a joint allocation of bandwidth and power in a complex wireless network scenario composed of network slicing and D2D communication since few works in the literature present resource allocation in mobile networks considering these two next-generation wireless network technologies. In this work, we propose an improvement to the DDPG algorithm using KNN and reward penalization to make its learning more efficient so that resource allocation is more adaptive to this new mobile communication scenario and meets the SLA requirements of each network slice.

### 4.3. Reducing the Action Space

The size of the action space in mobile network resource allocation considering the NS paradigm is very high, as it is proportional to the number of services in the network and the possible combinations of actions. The number of possible decisions exponentially increases with slots and services. Due to this huge action space, the BS will likely exploit undesirable actions during the training phase, such as allocating resources that cannot satisfy the data rate constraints and delay requirements or those exceeding the total amount of available resources, which will slow down the training convergence speed and prevent the DRL from maximizing the reward. In this work, we consider resource allocation in a simulation time of 1 s, that is, 2000 slots to be allocated among the two services, so the number of possible combinations for the two services will be $2000\times 2000=4\times 10^{6}$.

As in [11], for the DQN and DDQN algorithms, we use action elimination to solve the high-dimensionality problem of the action space. Action elimination is the technique of excluding combinations of undesirable actions from the space of actions in order to increase the velocity and quality of the training policy. After a sufficient number of training episodes, the elimination of undesirable actions allows the trained RL network (DQN and DDQN) in BS to generate good strategies (i.e., resource allocation decisions). Due to the neural network output of DQN and DDQN being composed of the number of possible combinations of actions, training the neural network in simulations becomes impractical without eliminating the undesired actions to reduce the action space.

To solve the problem of undesirable actions as in [11], we employ action elimination, a technique for excluding undesirable actions among all possible combinations of actions, increasing the speed and quality of the training policy. The algorithm consists of entering the total number of available resources (ActionMax), in this case, the maximum number of slots for allocation, which is 2000, as described in Section 3.2. It then determines the slot allocation combinations between the two services (URLLC and video) that exceed ActionMax, as shown in Algorithm 2. Due to the elimination of undesirable actions, after a reasonable number of training episodes, the agent makes resource allocation decisions that improve throughput and satisfy the SLA constraints of each service without exceeding the total amount of available resources.
**Algorithm 2:** Action elimination
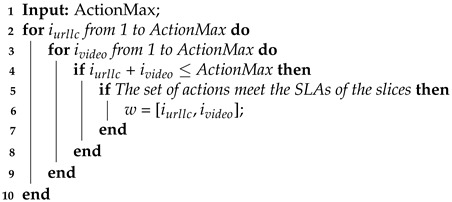



## 5. Evaluation Results

### 5.1. Experimental Design

In this section, we evaluate the DDPG-KRP algorithm performance with the network simulations with NS and D2D paradigms. In the simulations, we considered 1 BS and 7 network slices based on two categories of services (i.e., URLLC and video) with D2D communications. Table 2 shows each category of services with the specified number slice, UEs, And the D2D pair links are represented by a Poisson distribution. The simulations were performed using the following software and hardware configurations: MATLAB software version R2023b, 1.00 GHz Intel Core i5-1035G1 processor, and 16 GB RAM with no dedicated video card. The parameters for the mobile network scenario were similar to those used in [1,9,16]. Table 3 presents the full set of parameters for the simulations.

We implemented the DDPG-KRP algorithm to solve the resource allocation problem in each network slice. The actor neural network was implemented with four fully connected hidden layers with 256 neurons each and the leaky ReLU activation function. Meanwhile, the critic neural network was utilized considering three fully connected hidden layers with 128 neurons each and the leaky ReLU activation function. The learning rates for both the actor and critic networks were 0.01. The batch size was 256. The replay buffer (*R*) size was 10,000. The discount factor for the cumulative reward was 0.90.

The performance of the proposed DDPG-KRP algorithm was compared with those of two other DRL methods (i.e., DQN and DDQN) considering the same reward function proposed in Equation (16) and the action discretization using percentages and by also eliminating the undesirable actions for the DQN and DDQN algorithms. The neural network of the two methods was implemented with four fully connected hidden layers with 64 neurons in each and the leaky ReLU activation function. The hyperparameters were set with a 0.01 learning rate, 0.1 random action choice chance, 0.9 discount factor, 256 batch size, and 10,000 replay buffer size.

The hyperparameters of the DDQN and DDPG were found by simple search until the smallest value was obtained for the loss function given by Equations (21) and (24), respectively. Notice that in this work, we used the same neural network for the DDQN and DQN, so they have the same network hyperparameter value.

### 5.2. Results and Discussion

**Figure 5:**
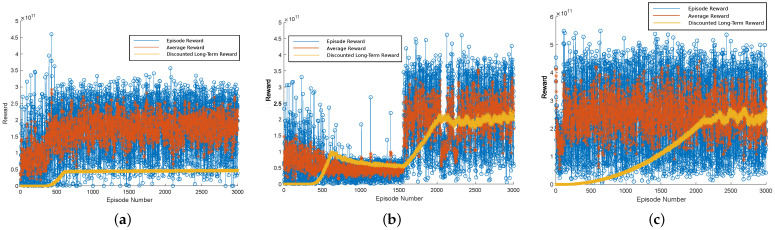
Reward comparison of different DRL agents: (**a**) DQN; (**b**) DDQN; and (**c**) DDPG-KRP.

After the agents performed the resource allocation for the slices, the power and bandwidth allocations were shared equally for the UEs in each slice. The performance of the RL algorithms was analyzed for over 3000 episodes in training. For a larger amount of episodes, we observed no significant changes in the results obtained with the considered agents. Figure 5a–c present the learning process of the agents in radio resource management. We plotted the reward values as a function of the number of episodes in the training phase. The sub-figures depict that the discounted long-term reward tends to gradually increase as the number of training episodes increases. The mean and immediate rewards presented random values due to the stochastic behavior of the environment. Figure 5a illustrates that the discounted long-term reward of the DQN agent becomes constant after 500 episodes due to the Q-value overestimation. In Figure 5b, the DDQN agent improves the result related to the other approaches by employing two evaluation functions in its learning method. In Figure 5c, DDPG-KRP shows faster convergence in training and higher reward values compared to the other considered approaches, which indicate better learning, as we expected, due to good learning ability in high-dimensional action space.

**Figure 6:**
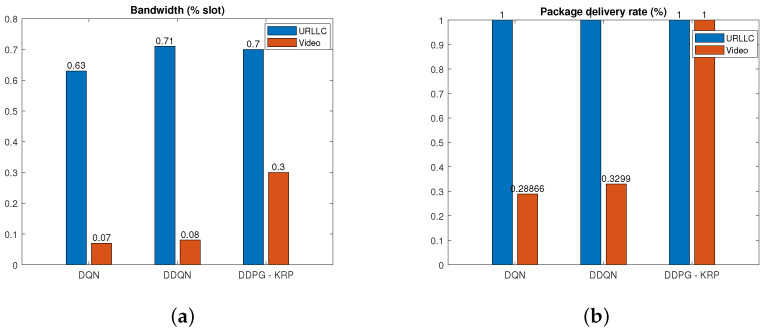
Performance comparison of different DRL agents for (**a**) bandwidth allocation. (**b**) PDR.

**Figure 7:**
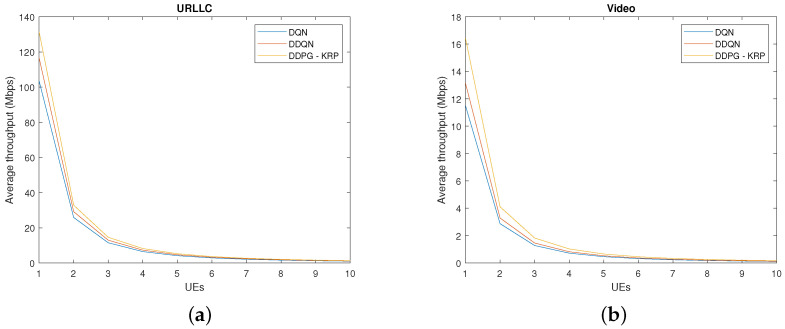
Performance comparison of different DRL agents for throughput and average delay in packet transmission: (**a**) URLLC slice throughput and (**b**) video slice throughput.

**Figure 8:**
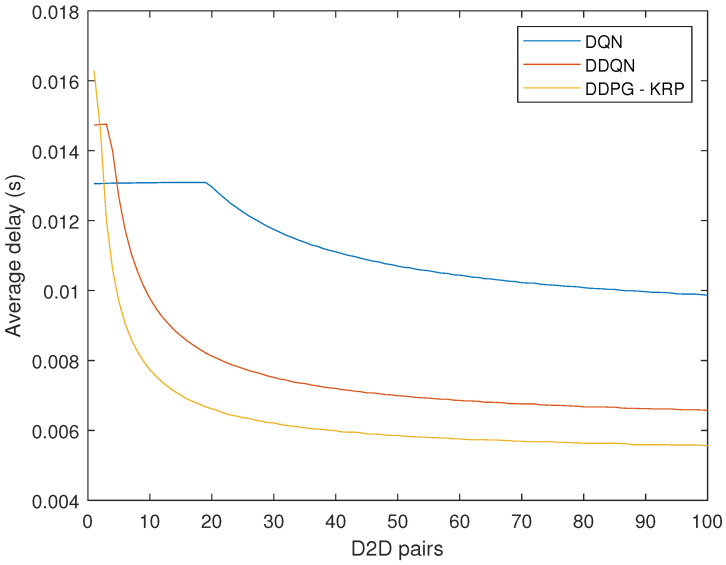
Average latency for each agent considering the two services.

**Figure 9:**
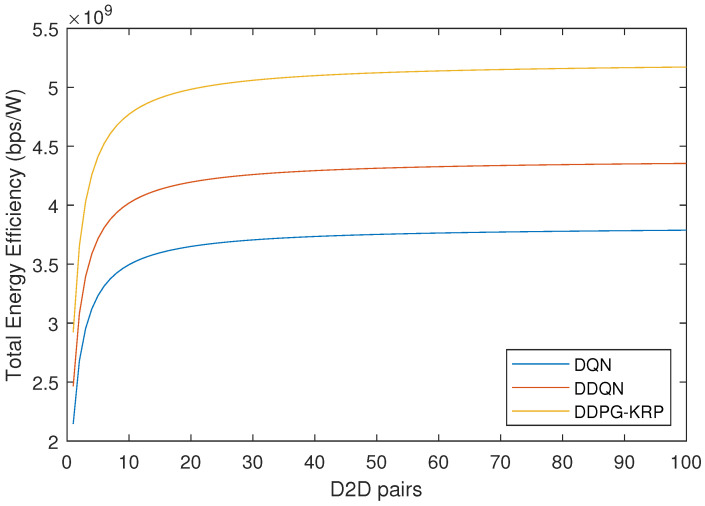
Energy efficiency by the number of D2D pairs for each agent.

**Figure 10:**
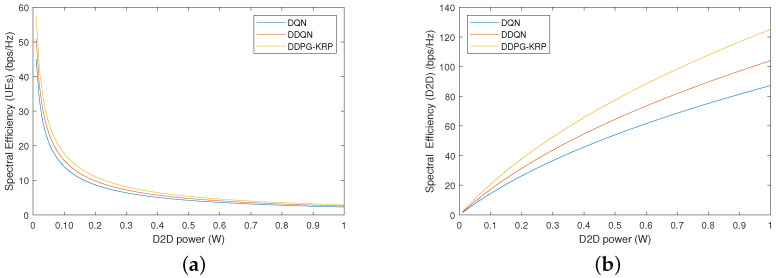
Spectral efficiency by the transmission signal power of the D2D pairs for each agent: (**a**) UEs; (**b**) D2D pairs.

Figure 6, Figure 7, Figure 8, Figure 9 and Figure 10 show a detailed comparison of the performance and results for the DRL agents after training. According to Table 2, a high weight β is assigned in the reward function to increase the number of digits and increase the priority of the PDR in each slice. Figure 6 shows that the resource allocation using the DQN algorithm does not allocate all available resources generating a low PDR. The DDQN algorithm can allocate almost all available resources but does not meet the PDR of all services. Compared to the DQN and DDQN algorithms, the proposed DDPG-KRP algorithm allows the agent to act by penalizing actions that do not meet the SLA requirements of each slice or by exceeding the total amount of available resources. DDPG-KRP generates a deterministic action in continuous space and then this action is discretized by the KNN algorithm. Figure 6 corroborates our belief that the DDPG-KRP presents better learning of the complicated relationship between demand and PDR than the other agents since all available resources are used and better performance in terms of the PDR is achieved compared to the other considered approaches.

Figure 7 compares the throughput by each DRL agent with the increase in UEs for each service. The traffic demands of the URLLC service are high because of the packet size. Given the higher transmission volume and the strictly lower latency requirement, the DRL agent learns to allocate a larger amount of resources to these slices, which shows a higher throughput. We observe that the proposed DDPG-KRP achieves a significant gain over the other methods. The highest throughput values for each service are because of its learning ability to allocate all available resources to the slices. We have applied a *t*-test to verify if the difference between the throughput values of Figure 7 for each approach is zero. We have found that the test does reject the null hypothesis at the 5% significance level.

The average delay is given by the average value for 1000 TTIs. Figure 8 compares the average delay values between the three DRL agents considering the complete system with all two services. The mathematical formulation of the average delay is not easy. Thus, the average delay is calculated based on a queue maintained in the BS, according to Equation (18). Figure 8 shows the better performance of the DDPG-KRP compared to the other algorithms. We have also applied a *t*-test to verify if the difference between the delay and throughput results of Figure 8 for each approach is zero. We have found that the test does reject the null hypothesis at the 1% significance level.

Figure 9 depicts that for the cellular wireless network, the increase in the number of D2D pairs causes an increase in the energy efficiency once increased throughput due to the spectrum reuse. To achieve good EE performance, devices must be able to communicate with each other using as little power as possible. This is a challenge for D2D communication because it often requires more transmission power than traditional communication methods [10]. The EE metric considered in this paper is given by Equation (17). In Figure 9, the DDPG-KRP shows the best result in terms of the EE.

Figure 10 shows the spectral efficiency (SE) value when varying the signal transmission power of the D2D pairs from 0 to 1 W. The remaining parameters stay the same. The results of Figure 10 are obtained by considering all two services. The SE metric is the ratio of the throughput to the allocated bandwidth. Figure 10b shows that the SE of the D2D pairs increases with the increasing signal transmission power. Accordingly, the highest value is achieved by the DDPG-KRP. However, in Figure 10a, the transmission signal power of the D2D pair should be increased in a controlled manner because it interferes with the UEs and degrades their performance requirements. The allocation of inappropriate resources and transmission power to the D2D pair introduces excessive interference, which could impair UE communication.

## 6. Conclusions

This work introduced an algorithm based on a DDPG agent, incorporating the KNN algorithm and reward penalization for undesirable actions, to address the resource allocation problem involving NS and D2D communication, taking into account the SLAs of each service. The mobile network reused the spectrum while considering the interference effects on resource allocation for UEs and D2D pairs. Lowering the transmit power of the D2D pairs reduced interference to the UEs, enabling the BS to also reduce its transmission power. Consequently, this resulted in power savings for both the BS and the D2D pairs.

Considering a huge number of possible combinations for resource allocation in each slice, the technique of eliminating the undesired actions was used to reduce the action space. Consequently, it eliminated approximately 85% of the undesired actions. Comparing the DDPG-KRP algorithm with the DRL-based techniques, namely the DQN and the DDQN, the extensive simulations showed that the DDPG-KRP can achieve better results due to the DRL-based approaches using the discrete action space, generating more delayed learning due to the large number of neurons in the deep neural network output.

Deep reinforcement learning methods are considered promising techniques for resource allocation in wireless networks. By monitoring the environment, these DRL methods provide interesting results without knowledge of the system statistics a priori because of their high learning potential. In this work, the DRL agent learns to allocate bandwidth and power by observing the network’s data traffic and information on the channel status of the UEs. The simulation results confirmed the learning effectiveness of the DDPG-KRP algorithm for allocating resources to UEs considering different services.

## Figures and Tables

**Table 1:** Notations.

Notations	Description	Notations	Description
Γ	Signal to interference plus noise ratio (SINR)	Q(s(t),a(t))	Q-value function
$\Gamma_{d}$	SINR of the D2D pair *d*	s(t)	System state
$\Gamma_{j}$	SINR of UE *j*	a(t)	System action
$G_{Bj}$	Channel gain between the BS and the UE *j*	r(s(t),a(t))	Reward function
$G_{jd}$	Channel gain between the UE *j* and the D2D pair *d*	α, β	Constants used to add weight to values and maintain them with the same number of digits
$G_{dd}$	Channel gain between the D2D pair *d* devices	$R_{b}$	Replay buffer
$G_{uv}$	Channel gain between two devices	PDR	Package delivery rate
$G_{Bd}$	Channel gain between the BS and the D2D pair *d*	NPR	Number of packets received
$p_{d}$	Transmission signal power from D2D pair *d*	NPQ(t)	Number of packets in the queue at time *t*
$p_{j}$	Transmission signal power from UE *j*	NPT	Number of packets transmitted
$\sigma_{z}^{2}$	Noise power	*R*	Discounted cumulative reward
$P_{bs}$	BS power	γ	discount factor
$d_{uv}$	Distance between the transmitter *u* and the receiver *v*	π(s)	Policy
$K_{uv}$	Normalization constant	$\pi {\left(s\right)}^{*}$	Optimal policy
δ	Loss exponent	$\mu (s|\theta^{\mu })$	Deterministic policy
PL	Path loss	*L*	Bellman loss function
$w_{d}$	Bandwidth of the D2D pair shared with the UE	*J*	Expected return from the start of the distribution
$w_{j}$	Bandwidth allocated to the UE *j*	*T*	Represents how many time steps are still left in the training session or episode
*Z*	Total number of UEs	η	Noise in the actor output
csi	Channel state information	ρ	Smoothing factor
*C*	Shannon capacity	$\Theta^{\prime }$	Weights of the target Q-network
L	Lagrange’s dual method	Θ	Weights of the main Q-network
λ	Lagrange multiplier	$\theta^{\mu }$	Weights of the actor main network
*E*[·]	Expectation operator	$\theta^{Q}$	Weights of the critic main network
∇	Gradient	$\theta^{\mu^{\prime }}$	Weights of the actor target network
EE	Energy efficiency	$\theta^{Q^{\prime }}$	Weights of the critic target network
*D*	Total number of D2D pairs	ActionMax	Total number of available slots for allocation

**Table 2:** Parameter settings.

Services	URLLC	Video
Slices	4	3
UEs	4	8
D2D pairs	Poisson distribution [mean = 5]	Poisson distribution [mean = 5]
Inter-arrival time distribution	Exponential [mean = 180 ms]	Truncated Pareto [exponential = 1.2, mean = 6 ms, max = 12.5 ms]
Packet size distribution	Truncated lognormal [mean = 2 MB, standard deviation = 0.722 MB, max = 5 MB]	Truncated pareto [exponential = 1.2, mean = 100 byte, max = 250 byte]
SLA: Rate	10 Mbps	5 Mbps
SLA: Latency	5 ms	10 ms

**Table 3:** Simulation parameters.

Parameters	Values
BS power ($P_{bs}$)	10 W
Transmission power D2D	0.1 W
Noise power	−114 dBm
Small cell radius	300 m
Maximum distance between a D2D pair	30 m
TTI	1 ms
Number of OFDM symbols per TTI	7
Simulation time	1000 TTI
RB bandwidth	180 KHz
Bandwidth	10 MHz
RBs per TTI	100
Mobility model	UEs and D2D pairs are uniformly distribute in the small cell to each TTI
Replay buffer ($R_{b}$)	10,000
Weight on channel capacity (α)	0.1
Weight on PDR (β)	10,000
Q-value update	1 s (2000 scheduling slots)

## Data Availability

Data are contained within the article.
